# The Prevalence of Nondiabetic Renal Diseases in Patients with Diabetes Mellitus in the University Hospital of Ribeirão Preto, São Paulo

**DOI:** 10.1155/2020/2129459

**Published:** 2020-06-13

**Authors:** Diego Agra Souza, Gyl Eanes Barros Silva, Igor Lima Fernandes, Dyego José Araújo de Brito, Monique Pereira Rêgo Muniz, Osvaldo Merege Vieira Neto, Roberto Silva Costa, Márcio Dantas, Miguel Moyses Neto

**Affiliations:** ^1^Pathology Service of the Ribeirão Preto Medical School Hospital, University of São Paulo, São Paulo, Brazil; ^2^Nephrology Division of the Hospital Presidente Dutra, Federal University of Maranhão, Maranhão, Brazil; ^3^Nephrology Division, Ribeirão Preto Medical School, University of São Paulo, São Paulo, Brazil; ^4^Laboratory of Renal Pathology, Nephrology Division, Ribeirão Preto Medical School, University of São Paulo, São Paulo, Brazil

## Abstract

**Objective:**

To evaluate the prevalence of nondiabetic renal diseases (NDRDs) in renal biopsies of patients with diabetes mellitus (DM) in the University Hospital of Ribeirão Preto, São Paulo. *Research Design and Methods*. We conducted a retrospective study including kidney biopsies performed in diabetic patients between 1987 and 2013. We evaluated 79 biopsies during this period. The primary variable was the prevalence of NDRD in patients with DM. The secondary variables were the presence of systemic arterial hypertension (SAH), hematuria, time since diagnosis of DM, serum creatinine, and proteinuria levels. The cases were divided into the following groups: isolated diabetic nephropathy (DN—group I), isolated nondiabetic renal diseases (NDRD—group II), associated NDRD/DN (group III), and associated NDRD+NDRD/DN (group IV).

**Results:**

Most of the patients (58.22%) presented only alterations arising from DN. NDRDs were present in 41.77% of the patients. Membranous glomerulonephritis (30.3%) and IgA nephropathy (24.24%) were the most prevalent NDRDs. We found no differences between female and male patients with NDRD when assessing the secondary variables. A time since diagnosis of five years or less revealed a statistical difference (*p* = 0.0005) in the comparison between the isolated DN (group I) and the NDRD+NDRD/DN (group IV). The other secondary variables were not significant in the comparison of the groups.

**Conclusions:**

We concluded that the prevalence of NDRD is 41.77%. Membranous glomerulonephritis was the most prevalent NDRD in our study. We also conclude that the probability of the presence of NDRD with or without concomitant DN is greater for patients who had biopsies with a time since diagnosis of five years or less. A time since diagnosis of ten years or more does not allow the exclusion of the presence of NDRD.

## 1. Introduction

Diabetic nephropathy (DN) is the leading cause of end-stage renal disease (ESRD) in developed countries. In the United States, more than 40% patients with ESRD have diabetes mellitus [1]. The Latin American countries which have the greatest prevalence of carriers of diabetes mellitus (DM) with ESRD are Puerto Rico (52.5%), Mexico (40%), Bolivia (36%), Chile (33%), and Colombia [2]. In Brazil, the estimated prevalence in the states São Paulo, Rio Grande do Sul, and Western Paraná is 9%, 26%, and 16.1%, respectively [3–5]. According to projections from the study by Wild et al. [6] in member countries of the World Health Organization, Brazil will have about 11.3 million diabetics by 2030, becoming the country with the eighth largest number of people affected by DM [6].

Although diabetic nephropathy is the leading cause of nephropathy in patients with DM, a wide variety of NDRDs may be present alone or coexist with DN. In recent years, there has been a great deal of discussion about biopsy indications for patients with DM, especially those with type 2. Generally, biopsies are performed for patients with diabetes mellitus type 2 who present with nephrotic-range proteinuria, renal failure in the absence of retinopathy or due to manifest nephropathy, unexplained glomerular hematuria, unexplained acute renal failure, and elevated creatinine clearance or serum albumin levels. However, due to the heterogeneity of lesions that may occur in concomitance with diabetes and the variability of the populations studied, biopsy indications for these patients still require more precision [7–12].

Studies which set out to identify the prevalence of nondiabetic renal diseases in diabetic patients found substantial variations ranging between 15% and 72.5% [13–20]. Moreover, there is considerable disagreement over which is the most common type of lesion. In Brazil, there are no studies that are aimed at determining which types of NDRD are present in patients with diabetes mellitus. We pose the following research question: what is the prevalence of NDRD in patients with diabetes mellitus who undergo biopsies in Ribeirão Preto? The aim of this study is to determine the prevalence of NDRD in renal biopsies of patients with diabetes in Ribeirão Preto, São Paulo.

## 2. Materials and Methods

This is a retrospective study that has been approved by the ethics committee of Ribeirão Preto Medical School of the University of São Paulo. The inclusion criteria were patients with DM who underwent renal biopsies between 1987 and 2013 and whose biopsies were evaluated by the respected Nephropathology Service of the University Hospital of Ribeirão Preto (University of São Paulo). The diagnostic criteria for DM were established by the American Diabetes Association (ADA) [21]. The exclusion criteria were biopsies of patients with transplanted diabetes mellitus and those with insufficient or unlocated clinical and laboratory information. We performed convenience sampling and reviewed the medical records of all the cases. Two experienced nephropathologists established the pathological diagnoses. We examined the cases with a light microscope with hematoxylin and eosin (2 sections), Masson's trichrome (2 sections), and periodic acid methenamine silver (2 sections) staining and direct immunofluorescence using fluorescein isothiocyanate (FITC) conjugated with antibodies for IgG, IgM, IgA, C1q, and fibrinogen. As evidence of diabetic nephropathy, we considered the presence of nodular or diffuse mesangial expansion, hyalinosis of afferent and efferent arterioles, thickening of the glomerular basement membrane, and the presence of exudative lesions (fibrin cap and capsular drop) [12, 22].

The primary variable was the prevalence of NDRD in patients with an established diagnosis of DM. Secondary variables were proteinuria and creatinine levels, the presence of hematuria and systemic arterial hypertension (SAH), a time since diagnosis of DM of ten years or more and a time since diagnosis of DM of five years or less. Complementary data included age and sex. We considered hematuria to be the presence of more than five red cells per higher-power field in the optical microscope. We compared group I (DN-only patients with alterations exclusively due to diabetic nephropathy), group II (NDRD-isolated nondiabetic renal diseases), group III (associated NDRD/DN) and group IV (NDRD+NDRD/DN-patients with isolated nondiabetic renal diseases and associated NDRD/DN). We also compared the differences between female and male patients in group IV.

We recorded the data in a spreadsheet (Microsoft Excel® 2010, Redmond, WA, USA). Descriptive statistical analysis was used to assess the complementary variables and data (95% confidence interval for each estimated point, prevalence, and mean). We applied the D'Agostino test of normality (proteinuria, creatinine, and age). The proteinuria and creatinine variables presented nonnormal distribution, while age presented normal distribution. To evaluate the data, we adopted the Mann-Whitney/Kruskal-Wallis test (proteinuria, creatinine), chi-square test with the Yates correction for continuity (presence of hypertension, hematuria, and time since diagnosis), and chi-square test for trend. We considered a *p* value of less than 0.05 to be significant. We used QuickCalcs (©2016 GraphPad Software) and Biostat (©2014 AnalystSoft Inc.) to calculate the data.

## 3. Results

During the period in question, the Nephropathology Pathology Service received 96 renal biopsies of diabetic patients. We excluded 17 of these cases either because they did not present sufficient clinical information or because we did not find clinical data and laboratory tests in the medical records. Thirty-nine patients (49.37%, 95% CI: 38.63%–60.16%) who underwent biopsies were female and forty (50.63%, 95% CI: 39.84%–61.37%) were male, making up a total of seventy-nine cases. Twenty-six male patients and twenty female patients presented only with alterations resulting from DN, totaling forty-six (58.22%, 95% CI: 47.21%-68.48%). Fourteen male patients and nineteen female patients presented with NDRD, totaling thirty-three (41.77%, 95% CI: 31.52%-52.79%). Of these, six cases (7.6%) presented with the concomitance of DN and other NDRDs (group III). Descriptive statistics of group III are shown in Table 1.

The main NDRDs we observed were membranous glomerulonephritis (10 cases), IgA nephropathy (8 cases), focal segmental glomerulosclerosis (4 cases), lupus nephritis (3 cases), and minimal change disease (2 cases). We described the other diseases in Table 2.

The mean age of the group of patients with only DN (group I) is 51.11 years (95% CI: 47.08-55.15), while it is 51.06 years (95% CI: 46.52-55.60) in group IV (NDRD+NDRD/DN). There was no statistically significant difference between the two groups regarding age (*p* = 0.929).

When comparing only the variables between men and women with NDRD+NDRD/DN (group IV), we found that the mean age for female patients was 49.44 years (95% CI: 45.00-53.87) and 52.89 years (95% CI: 49.14-56.65) for male patients. There was no statistically significant difference (*p* = 0.2399). Among men, 35% had nondiabetic renal diseases, while the proportion among women was 48.71%. There was no statistical difference between the proportions of men and women affected by NDRD (*p* = 0.2164). We also compared the isolated NDRD (group II), DN (group I), concomitant NDRD and DN groups (group III) and NDRD+ NDRD/DN (group IV), as shown in Tables 1 and 3.

The remaining variables (time since diagnosis of five years or less, time since diagnosis of ten years or more, presence of associated systemic arterial hypertension, and presence of hematuria, creatinine, and proteinuria values) are illustrated in Tables 1 and 3. There was no statistically significant difference between male and female groups.

We found exams indicating diabetic retinopathy for three patients in the NDRD group (group IV). Three patients in group IV and five patients in group I presented with diabetes mellitus type 1; the others presented with type 2 diabetes.

## 4. Discussion

Many studies have set out to establish criteria for renal biopsies of patients with diabetes mellitus, especially diabetes type 2. Attention is drawn to the significant variation in the prevalence of NDRD, which ranges between 15% and 93.5% [10]. These variations can be explained by epidemiological factors, different clinical criteria for biopsy indications and the adoption of different inclusion criteria in studies. In the literature, Zhuo et al. [18] reported the highest prevalence of NDRD in diabetic patients (93.5%) and used the presence of microalbuminuria within the first five years following diagnosis as one of the clinical criteria. In our study, the main reasons why diabetic patients underwent biopsies were the presence of proteinuria, signs of acute renal failure, the presence of associated autoimmune diseases (e.g., lupus), and hematuria. We demonstrated that 41.77% of the patients presented with NDRD+NDRD+DN (group IV). In spite of the considerable variation in the literature, this prevalence is important given the lack of research on this matter in Brazil and Latin America.

The presence of diabetic retinopathy is usually cited as a factor in favor of the diagnosis of DN. In some studies, the presence of diabetic retinopathy was used as a criterion to limit the biopsy [8, 19]. Some researchers found a statistically significant association between diabetic retinopathy and diabetic nephropathy [9, 14]. However, it is important to note that the presence of diabetic retinopathy does not exclude the existence of NDRD [7]. In this study, the presence of retinopathy was a limiting factor, as it was not always described in the medical records. As this is a retrospective study which covered an extensive period (26 years), it was difficult to locate some files. In addition, some biopsies sent to the hospital's nephrology service came from external medical services with requests that presented little clinical information, which made it difficult to find some data. All of these factors resulted in the exclusion of some cases.

We chose to compare and evaluate two or three groups. Most researchers choose this distribution of groups. Zhuo et al. established three groups based on three age ranges [19]. Wilfred et al. found male patients to be the majority in the NDRD/DN and DN groups [12]. However, we did not find studies that described differences between the genders comparing the variables time since diagnosis and proteinuria. We observed a more discreet number of women with NDRD, but there was no statistical difference. The presence of hematuria, SAH, and proteinuria and creatinine levels did not reveal statistical differences in the groups. Lin et al. [7] did not find significant differences when studying the presence of hematuria in groups with and without NDRD. Most studies did not find differences among the groups concerning the variables proteinuria [9, 12–14] and serum creatinine levels [7, 9, 12, 14].

Although the prevalence of SAH is higher in the DN group, there was no statistical difference in the comparison of DN (group I) and isolated NDRD (group II) or DN (group I) and NDRD+NDRD/DN (group IV). In the literature, the association of hypertension and DN has controversial results [9, 12, 14]. A time since diagnosis of more than ten years did not present a statistical difference when compared to the NDRD+NDRD/DN group (group IV). This finding is compatible with the findings of Lin et al. [7] who concluded that a time since diagnosis of DM over ten years does not allow the exclusion of NDRD. When we compared the cases with a time that was five years or less, we concluded that most of the cases (70.37%) represent NDRD (*p* < 0.05). In favor of this finding, we highlight that Zhuo et al. [18] found a prevalence of 93.5% in patients who underwent biopsies and had a time since diagnosis of 5 years or less. Tone et al. [23] concluded that the absence of retinopathy and a time since diagnosis of less than five years are useful guides in making the decision to perform a biopsy in a diabetic patient with proteinuria. Even though most of the patients who underwent biopsies and had a time since diagnosis of less than five years presented with NDRD, we emphasize that a little less than one-third of the patients within this period presented with isolated DN (group I).

As for the prevalence of NDRD, we observed membranous glomerulonephritis to be the most common, followed by IgA nephropathy. The results of other studies with respect to the most prevalent NDRDs vary: postinfectious glomerulonephritis [13], IgA nephropathy [9, 15, 19, 24, 25], acute tubular necrosis [20], focal segmental glomerulosclerosis [26], and membranous glomerulonephritis [27, 28]. Many studies on the frequency of glomerulopathies have highlighted focal segmental glomerulosclerosis as the most frequent. In Latin America, lupus nephritis is a more frequent glomerulopathy. Attention is drawn in the United States and Canada, and the number of cases of diabetic glomerulosclerosis is practically the same as that of focal segmental glomerulosclerosis. This reflects differences between populations and the criteria for performing a biopsy of patients with diabetes. We emphasize that these studies found a frequency of all glomerulopathies and were not dedicated to describing the findings of nondiabetic kidney disease in patients diagnosed with diabetes [29–31]. As described in other studies with a similar theme, the number of cases of diabetic patients biopsied was limited. We analyzed cases from a region in the state of São Paulo, and the data cannot be extrapolated to the whole of Brazil, which has a very vast territorial extension and a wide ethnic and socioeconomic variation. We emphasize that this study does not reflect a real prevalence of NDRD in patients with DM. This is because there is a bias in the biopsy indication, since clinicians performed biopsies based on their own clinical indications.

## 5. Conclusion

We concluded that the prevalence of NDRD in Ribeirão Preto is 41.77% and that there is a higher probability of NDRD in proteinuric patients with a time since diagnosis of diabetes mellitus of less than 5 years. In our study, creatinine and proteinuria levels and the presence of hematuria and hypertension proved not to be of use in making the distinction between NDRD and DN. Given that numerous studies demonstrate diverse results, it is necessary to carry out a global, prospective study with well-defined inclusion criteria. The decision as for when to perform a renal biopsy in a diabetic patient still remains a challenge.

## Figures and Tables

**Table 1:** Sample description and comparison between groups DN (group I)/isolated NDRD (group II)/associated NDRD and DN (group III).

	Isolated DN (group I)	Isolated NDRD (group II)	Associated DN and NDRD (group III)	*p* value
Time ≥ 10 years	41.3% (28.27%-55.68%, CI 95%)	18.52% (7.72%-37.16%, CI 95%)	33.33 (9.25%-70.43%, CI 95%, CI 95%)	0.1606^∗^
Hematuria	45.65% (32.15%-59.82%, CI 95%)	55.56% (37.30%-72.43%, CI 95%)	50% (18.76%-81.14%, CI 95%)	0.5656^∗^
SAH	76.09% (61.91%-86.24%, CI 95%)	62.96% (44.16%-78.53%, CI 95%)	33.33 (9.25%-70.43%, CI 95%)	0.3534^∗^
Proteinuria	4.48 (4.21–6.93, CI 95%)	3.36 (3.39–6.06, CI 95%)	2.36 (2.91–7.87, CI 95%)	0.6562
Creatinine	2.41 (1.94–3.40, CI 95%)	1.24 (1.29–2.28, CI 95%)	1.83 (0.87–4.72, CI 95%)	0.495
Age	51.11 (47.08-55.15, CI 95%)	51.44 (46.20-56.69, CI 95%)	49.33 (37.37-61.29, CI 95%)	0.9860
Sex	26 males/20 females	11 males/16 females	3 males/3 females	—

^∗^DN (group I) and isolated NDRD (group II).

**Table 2:** Glomerular disease diagnoses.

	Men	Women	Total
Isolated nondiabetic renal diseases			
Membranous glomerulonephritis	4	6	10 (30.3%, 95% CI 17.25%-47.46%)
IgA nephropathy	4	4	8 (24.24%, 95% CI 12.60%-41.25%)
Lupus nephritis	—	3	3 (9.09%, 95% CI 2.37%-24.34%)
Focal segmental glomerulosclerosis	2	1	3 (9.09%, 95% CI 2.37%-24.34%)
Minimal change disease	—	1	1 (3.03%, 95% CI 0.01%-16.65%)
Benign nephrosclerosis/chronic tubulointerstitial nephritis	—	1	1 (3.03%, 95% CI 0.01%-16.65%)
Chronic tubulointerstitial nephritis	1	—	1 (3.03%, 95% CI 0.01%-16.65%)
Nondiabetic renal diseases associated with diabetic nephropathy			
Nephropathy associated with HIV+diabetic nephropathy	—	1	1 (3.03%, 95% CI 0.01%-16.65%)
Diffuse proliferative glomerulonephritis+diabetic nephropathy	—	1	1 (3.03%, 95% CI 0.01%-16.65%)
Focal segmental glomerulosclerosis+diabetic nephropathy	—	1	1 (3.03%, 95% CI 0.01%-16.65%)
Glomerulonephritis associated with unclassified immune complexes+diabetic nephropathy	1	—	1 (3.03%, 95% CI 0.01%-16.65%)
Minimal change disease+diabetic nephropathy	1	—	1 (3.03%, 95% CI 0.01%-16.65%)
Postinfectious glomerulonephritis+diabetic nephropathy	1	—	1 (3.03%, 95% CI 0.01%-16.65%)
Total	14	19	33

95% CI: 95% confidence interval; HIV: human immunodeficiency virus.

**Table 3:** Comparison between groups DN (group I) and NDRD+NDRD/DN (group IV).

	Isolated DN (group I)	NDRD+NDRD/DN (group IV)	*p* value
Time ≤ 5 years	17.39% (8.82%-30.99%, CI 95%)	57.58% (40.79%-72.78%, CI 95%)	0.0005
Time ≥ 10 years	41.30% (28.27%-55.68%, CI 95%)	21.21% (10.38%-38.05%, CI 95%)	0.1028
Hematuria	45.65% (32.15%-59.82%, CI 95%)	54.55% (37.98%-70.16%, CI 95%)	0.5812
SAH	76.09% (61.91%-86.24%, CI 95%)	57.58% (40.79%-72.78%, CI 95%)	0.1388
Proteinuria	4.48 (4.21-6.93, CI 95%)	3.18 (3.33–5.98, CI 95%)	0.9555
Creatinine	2.41 (1.94–3.40, CI 95%)	1.39 (1.47–2.46, CI 95%)	0.378
Age	51.11 (47.08-55.15, CI 95%)	51.06 (46.52-55.60, CI 95%)	0.929
Sex	26 males/20 females	14 males/19 females	—

## Data Availability

Data supporting study conclusions are included within this report. Additional data are available from the corresponding author upon reasonable request and with permission from the Nephropathology Service of the University Hospital of Ribeirão Preto.
